# Gastric Intestinal Metaplasia in an Underserved Population in the USA: Prevalence, Epidemiologic and Clinical Features

**DOI:** 10.1155/2013/856256

**Published:** 2013-10-22

**Authors:** Tarek Almouradi, Tadd Hiatt, Bashar Attar

**Affiliations:** Division of Gastroenterology and Hepatology, Department of Medicine, John H. Stroger Hospital of Cook County, 1901 W. Harrison Street Admin. bldg #1439, Chicago, IL 60612, USA

## Abstract

Gastric intestinal metaplasia is an important stage in the development of gastric cancer. Limited data is available regarding the prevalence of gastric intestinal metaplasia in the United States. We conducted a retrospective review of esophagogastroduodenoscopies performed in our endoscopy unit between the months of April and October 2010 to evaluate the prevalence and the epidemiologic and endoscopic features of gastric intestinal metaplasia in an underserved population in the United States.

## 1. Introduction

Gastric cancer is a global health problem, the second leading cause of cancer related mortality worldwide, and is responsible for about 740,000 yearly deaths [1]. In the United States alone, about 21,600 adults (13,230 men and 8,370 women) will be diagnosed with stomach cancer in 2013. In the same year, gastric cancer will be responsible for an estimated 10,990 deaths [2]. Of note, the incidence rate of gastric cancer in the United States differs by ethnicity, with the highest incidence in Hispanics (12 in 100,000) and African Americans (11 in 100,000) compared to 6 in 100,000 in Caucasians [3].

The pathogenesis of gastric adenocarcinoma, described by Dr. Pelayo Correa as the gastric precancerous cascade, involves a multistep progression from chronic gastritis to atrophy, intestinal metaplasia, dysplasia, and ultimately neoplasia [4]. Despite the importance of gastric intestinal metaplasia as a precancerous condition, limited data is available regarding its prevalence in the United States.

We aimed to evaluate the prevalence, epidemiologic, and clinical features of gastric intestinal metaplasia in an underserved population in the United States.

## 2. Methods

This was a retrospective review of esophagogastroduodenoscopies performed in the Endoscopy Unit at John H. Stroger Hospital of Cook County (a large public hospital in Chicago, IL, USA) between the months of April and October, 2010. Data including age, gender, indication for endoscopy, and endoscopic findings were retrieved from the computerized endoscopy reporting system (Olympus EndoWorks version 7.4.42.16). Then, we performed a retrospective chart review to obtain data regarding ethnicity, histological findings (when biopsies were performed), and *Helicobacter pylori* infection status (determined by histological examination of biopsies and/or rapid urease test). In patients who underwent multiple esophagogastroduodenoscopies within the given time period, we only included results from the first test. The Institutional Review Board of our hospital approved the study.

## 3. Histology

Biopsy specimens were placed in vials containing 10% buffered formalin solution. Paraffin sections were prepared and stained using standard Hematoxylin and Eosin staining procedures. Our pathologists, who are experienced in detecting gastric histological abnormalities and determining the presence of gastric intestinal metaplasia, reviewed the pathology slides. The presence of active *Helicobacter pylori* infection was determined by a positive CLO test (rapid urease test by Kimberly-Clark) and/or detection of *H. pylori* organisms in Immunostained gastric biopsies.

## 4. Statistical Methods

All statistical analyses were performed using Epi Info 6, by the Centers for Disease Control and Prevention (CDC). The demographic and clinical characteristics of the patients were compared by chi-square testing, and *P* values of <0.05 were considered significant. We also used the chi-square test to evaluate whether there is a linear trend between age and different variables.

## 5. Results

677 patients (363 females and 314 males) underwent esophagogastroduodenoscopy during the set time period. The majority of patients were African Americans (*n* = 291) and Hispanics (*n* = 273) compared to Caucasians (*n* = 65) and Asians (*n* = 48). Patient demographics are presented in Table 1. The main indication for upper endoscopy was dyspepsia (274 cases), anemia (96 cases), and upper GI bleeding such as hematemesis and melena (83 cases). Indications for esophagogastroduodenoscopy are presented in Table 2. In 200 of the 677 cases, upper endoscopy showed normal gastric mucosa. Pathological findings included nonerosive and atrophic gastritis (169 cases), gastric erosions (153 cases), and gastric ulcers (47 cases). Endoscopic findings are shown in Table 3. *Helicobacter pylori* infection was checked in 600 patients. The prevalence of active infection was 43%.

## 6. Gastric Intestinal Metaplasia

Among the 437 patients who had gastric biopsies performed, 66 were found to have gastric intestinal metaplasia. The overall prevalence was 15%. We found a statistically significant female predominance, with prevalence of 18.5% (42 of 226) compared to 11% in males (24 of 211) (*P* 0.035).

**Figure 1:**
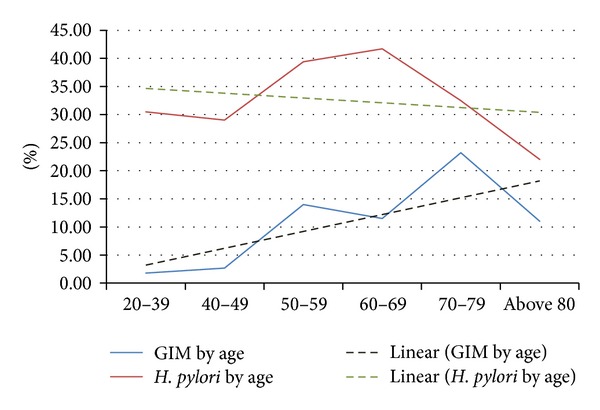
Trend of gastric intestinal metaplasia and active *Helicobacter pylori* with age.

We also observed a significant increase in the prevalence of gastric intestinal metaplasia with age (chi-square for linear trend 12.86, *P* value <0.001), although we did not observe a similar increase in the prevalence of active *Helicobacter pylori* infection (chi-square for linear trend 1.9, *P* value 0.16) (Figure 1). Of note, there was no statistically significant difference in the prevalence of gastric intestinal metaplasia in individuals with active *H. pylori* infection compared to uninfected individuals (16.8% compared to 13%, *P* = 0.27).

**Figure 2:**
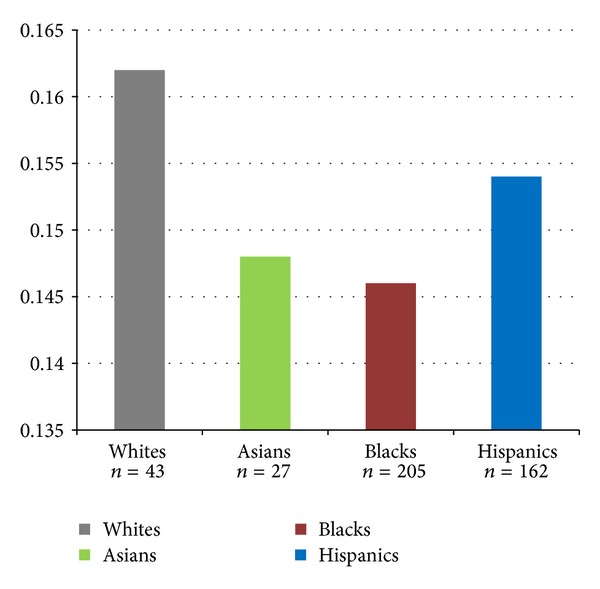
Prevalence of gastric intestinal metaplasia in different ethnic groups.

The highest prevalence of gastric intestinal metaplasia was found among Caucasians (16.2%), followed by Hispanics (15.4%), Asians (14.8%), and African Americans (14.6%) These findings, however, were not statistically significant (*P* = 0.99).  Figure 2 represents the prevalence of gastric intestinal metaplasia in the various ethnic groups.

In regard to the indications of esophagogastroduodenoscopy, the prevalence of gastric intestinal metaplasia among patients undergoing endoscopy to evaluate the source of upper GI bleeding was 20% (10 out of 50), compared to 16.6% in patients being worked up for anemia (12 out of 72) and 15.6% in patients evaluated for dyspepsia (27 out of 173). No statistical significance was noted (*P* = 0.76).

In regard to endoscopic findings, gastric intestinal metaplasia was found in 24% of patients who had gastric ulcers (11 of 45), compared to 16% in patients with erosive gastritis (21 of 132) and nonerosive or Atrophic gastritis (24 of 150). These findings were also not statistically significant (*P* = 0.37).

## 7. Discussion

Based on our review of the literature, very limited data is available regarding the prevalence of gastric intestinal metaplasia in the United States. A study performed in 1992 by Fennerty et al., represents the only such study. They aimed to establish the prevalence of intestinal metaplasia and to identify associated epidemiological factors in the southwestern United States [5]. They included 440 patients from the Veterans Administration Medical Center in Tucson, Arizona, USA. The vast majority of the patients were males, with only two females included. Unlike our study, their study population consisted mainly of whites (*n* = 359). They found the overall prevalence of gastric intestinal metaplasia to be 19%, which was close to the prevalence we are reporting (15%). Similar to our study, they noted a significant trend of gastric intestinal metaplasia with age, with no demonstrable age trend in regard to the presence of *H. pylori* infection. But unlike our study, they found significant differences in the prevalence of gastric intestinal metaplasia between different ethnic groups (50% in blacks and Hispanics compared to 13% in whites). Of note, most of the published data regarding the prevalence of gastric intestinal metaplasia comes from Europe and Asia, with the reported prevalence ranging between 8.9% and 38%.

Some of the more robust prevalence numbers come from studies performed in Europe. A study by Craanen et al. from the Netherlands showed a 25.3% overall prevalence of gastric intestinal metaplasia in patients undergoing upper endoscopy for various indications between December 1988 and June 1990. When dividing patients into two age groups, gastric intestinal metaplasia was found significantly more often in patients 50 years of age or older, compared to patients younger than 50 (31.9% versus 10.4%). The highest prevalence was noted in patients older than 80 (46.6%) [6]. In Germany, Eidt and Stolte reported a 25.7% prevalence of gastric intestinal metaplasia in 1446 *H. pylori* infected patients. Unlike our study, they reported significantly higher prevalence in patients who had gastric ulcers compared to other endoscopic findings [7]. In The Netherlands, Tulassay et al. [8] conducted a multicenter study to evaluate endoscopic and histological findings after *Helicobacter pylori* eradication therapy in 401 patients with gastric ulcers. They reported a baseline prevalence of gastric intestinal metaplasia in antral biopsies ranging from 27.5% to 38.1%. In Sweden, Petersson et al. found gastric intestinal metaplasia in 23% of gastric biopsies from 475 subjects randomly selected from the general population [9]. Multiple studies from Italy reported variable prevalence rates of gastric intestinal metaplasia. Vaira et al. reported prevalence of 11–16% in 300 patients with dyspepsia and *H. pylori* infection [10]. Another study published in 2010 found gastric intestinal metaplasia in 15.5 percent of patients infected with *H. pylori* infection [11]. Similarly, Scaccianoce et al. found gastric intestinal metaplasia in 19% of 213 patients with dyspepsia, and *H. pylori* infection enrolled in their study to evaluate different *H. pylori* therapeutic regimens [12]. Of note, the highest reported prevalence of gastric intestinal metaplasia in the reviewed studies from Italy was 32% in elderly patients (mean age of 69.5) with peptic ulcer disease and *H. pylori* infection [13].

In reviewing published studies from Asia, we noted prevalence rates ranging from 8.9% to 37%. In their study published in 2009 from Hong Kong, Yee et al. reported a 9.4% prevalence of gastric intestinal metaplasia. They also noted an increase in prevalence with age, with the highest prevalence of 25% in patients older than 60. But unlike our study, they did not find a significant difference in regard to gender [14]. In 2004, Wong et al. aimed to determine whether the treatment of *H. pylori* infection reduces the risk of gastric cancer in a Chinese population. They reported the gastric intestinal metaplasia prevalence to be 29.3% in 1630 healthy carriers of *H. pylori* infection [15]. In Malaysia, where the reported prevalence of *H. pylori* is low (4.2%), Yeh et al. reported a 9.8% prevalence of gastric intestinal metaplasia in the examined biopsies of patients undergoing upper endoscopy for various indications [16]. Lastly, in a Japanese study aimed to evaluate the role of *H. pylori* infection in the development of gastric cancer, Uemura et al. found gastric intestinal metaplasia in 37% of *H. pylori* infected individuals compared to only 2% of uninfected individuals, a difference we did not observe in our population. It is worth mentioning that our population has a much lower prevalence of active *H. pylori* infection (43% compared to 81.6%) [17].

In summary, gastric intestinal metaplasia is a common finding in patients undergoing diagnostic upper esophagogastroduodenoscopy. We noted a significant increase in the prevalence with age, however, without a similar increase in active *H. pylori* infection. In addition, we also noted a higher prevalence in females, but not between different ethnic groups. Larger epidemiologic studies will be helpful to confirm our findings and to help guide endoscopic screening toward specific groups at risk for gastric cancer in the United States.

## Figures and Tables

**Table 1:** Demographic characteristics of the study population (total number 677).

Gender	
Females	363 (53.6%)
Males	314 (46.5%)
Age group	
19–29	39 (5.7%)
30–39	69 (10%)
40–49	144 (21%)
50–59	233 (34.4%)
60–69	140 (20.6)
70–79	43 (6.3)
Above 80	9 (1.4%)
Race/ethnicity	
Black	291 (43%)
Hispanic	273 (40%)
White	65 (10%)
Asian	48 (7%)

**Table 2:** Indication for esophagogastroduodenoscopy.

Dyspepsia	274 (40%)
Workup for anemia	96 (14%)
Overt GI bleeding	83 (12%)
GERD	75 (11%)
Dysphagia	44 (6.5%)
Screening/surveillance for esophageal varices	43 (6%)
Other^∆^	62 (10%)

^∆^Other indications include abdominal pain, diarrhea, nausea, vomiting, and abnormal imaging.

**Table 3:** Endoscopic findings of gastric evaluation.

Normal	200 (29.97%)
Nonerosive gastritis/atrophic gastritis	169 (24.9%)
erosions/Erosive gastritis	153 (22.5%)
Ulcer (gastric/duodenal)	47 (6.9%)
Other findings^∃^	108 (15.9%)

^∃^Other findings include mucosal nodularity, gastric polyps, masses, and visible vessels/Dieulafoys lesions.
